# Decoding potential targets and pharmacologic mechanisms of curcumin in treating non-small cell lung carcinoma via bioinformatics and molecular docking

**DOI:** 10.1590/1414-431X2024e13550

**Published:** 2024-09-06

**Authors:** Jie Li, Zhen Zhang, Junchang Zhao, Shilin Liu, Chenghong Feng, Hong Deng, Dongwen Liu, Jing Zeng, Qin Yu, Dan Zhou, Milin Zhu, Yantao Liu

**Affiliations:** 1 Department of Pharmacy, West China Second University Hospital, Sichuan University, Chengdu, China; 2 Key Laboratory of Birth Defects and Related Diseases of Women and Children, Sichuan University, Ministry of Education, Chengdu, China; 3 School of First Clinical Medical College, Mudanjiang Medical University, Mudanjiang, China; 4 Postgraduate Department, Mudanjiang Medical University, Mudanjiang, China

**Keywords:** Curcumin, NSCLC, Network pharmacology, Pharmacologic mechanisms, Bioinformatics

## Abstract

Emerging evidence demonstrates that curcumin has an inhibitory effect on non-small cell lung cancer (NSCLC), and its targets and mechanism of action need further exploration. The goal of this study was to explore the potential targets and mechanism of curcumin against NSCLC by network pharmacology, bioinformatics, and experimental validation, thereby providing more insight into combination treatment with curcumin for NSCLC in preclinical and clinical research. Curcumin targets against NSCLC were predicted based on HIT2.0, STD, CTD, and DisGeNET, and the core targets were analyzed via protein-protein interaction network construction (PPI), Gene Ontology (GO), Kyoto Encyclopedia of Genes and Genomes (KEGG), and molecular docking. The gene expression levels of samples in A549 cells, NCI-H460, and curcumin treated groups were detected by real-time quantitative PCR. A total of 67 common targets between curcumin and NSCLC were collected by screening public databases. GO and KEGG analysis suggested that curcumin treatment of NSCLC mainly involves cancer-related pathways, such as PI3K-AKT signaling pathway, Foxo signaling pathway, microRNAs, MAPK signaling pathway, HIF-1 signaling pathway, etc. The targets with the highest degree were identified through the PPI network, namely CASP3, CTNNB1, JUN, IL6, MAPK3, HIF1A, STAT3, AKT1, TP53, CCND1, VEGFA, and EGFR. The results of the *in vitro* experiments showed that curcumin treatment of NSCLC down-regulated the gene expressions of *CCND1*, *CASP3*, *HIF1A*, *IL-6*, *MAPK3*, *STAT3*, *AKT1*, and *TP53*. Our findings revealed that curcumin functions as a potential therapeutic candidate for NSCLC by suppressing multiple signaling pathways and interacting with multiple gene targets.

## Introduction

Lung cancer is one of the most common malignant tumors with high worldwide morbidity and mortality, accounting for approximately 27% of all carcinoma-associated deaths, with an incidence rate of 14.5% in men and 8.4% in women (1). Its pathogenesis is complicated, and smoking or long-term exposure to cigarette carcinogens are identified as risk factors, which can induce bronchial mucosal or gland lesions, thereby resulting in the formation of lung neoplasms (2). In addition, numerous other hazard factors have been recognized as related to lung tumor etiology, such as age, environmental pollution, family history, decreased immunity, chronic obstructive pneumonia, and infection (3).

Lung cancer has been classified into two types according to its histopathologic category, namely non-small cell lung cancer (NSCLC) and small cell lung cancer (SCLC), in which NSCLC accounts for about 85% of lung cancers, including adenocarcinoma (approximately 40%), squamous cell carcinoma, (25 to 30%), and large cell carcinomas (10 to 15%) (4). However, some specialists suggest that abandoning the existing classifications of NSCLC may be critical in developing novel, more effective treatment strategies (5). Currently, clinical treatments for NSCLC involve chemotherapy, immunotherapy, targeted therapy, and combined therapy in addition to the most effective surgery (6). However, the long-term use of these drugs may induce resistance (7). Additionally, the etiology and pathogenesis of NSCLC are complicated. Therefore, it is urgent to continuously develop more effective agents or adjuvants for eradicating NSCLC.

**Figure 1:**
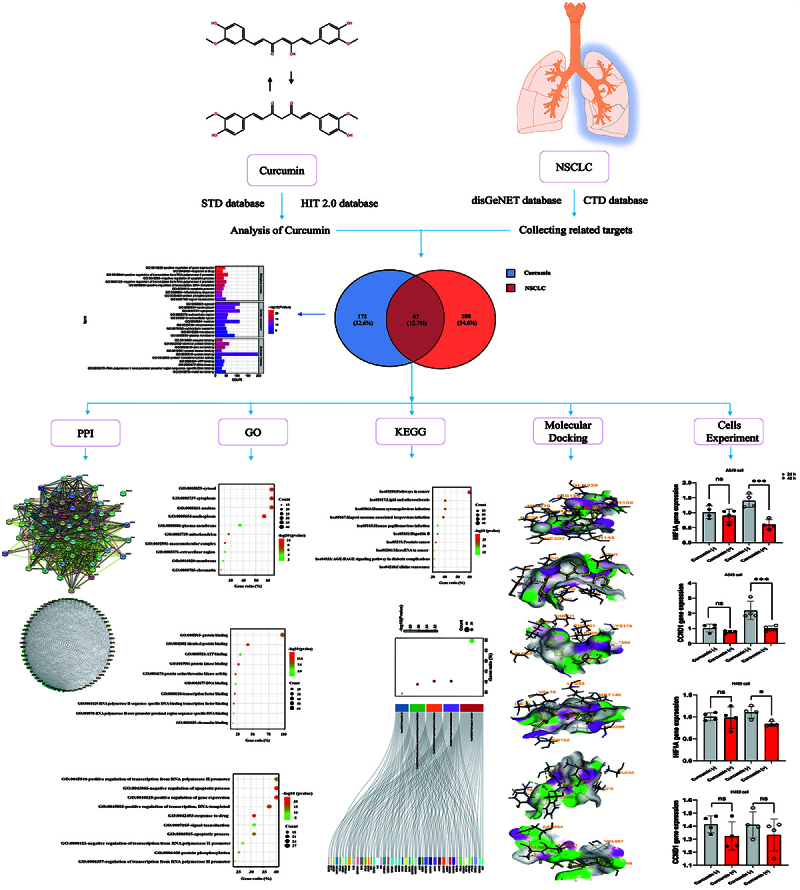
Flow diagram of investigations of the mechanisms of curcumin against non-small cell lung cancer (NSCLC). PPI: protein-protein interaction network construction, GO: Gene Ontology, KEGG: Kyoto Encyclopedia of Genes and Genomes.

Curcumin, as a natural small molecule compound, has a variety of pharmacological and biological activities, such as anti-oxidation (8), anti-inflammatory and anticancer (9), anti-infection (antibacterial, antifungal, or anti-viral) (10), anti-senescence (11), reducing blood glucose and blood lipids (12), and extending life span (13), etc. Most curcumin studies mainly focus on the investigation of cancer, especially lung and breast carcinomas. Preclinical studies demonstrate that curcumin exhibits an excellent inhibitory effect on NSCLC by inducing oxidative stress (14), inhibiting cell proliferation and invasion (15), promoting cell apoptosis (16), and partially regulating immune system (17). Unfortunately, these anticancer effects are observed in animals or cells and clinical research is relatively scarce. Utilizing bioinformatics, network pharmacology, and experimental verification, this study was designed to investigate the multiple targets and pathways of curcumin against NSCLC and provide more insight into drug resistance and combination drugs, as well as preclinical and clinical research. The workflow is shown in Figure 1.

## Material and Methods

### Collecting the targets of curcumin

In this study, Herbal Ingredients' Targets platform-HIT 2.0 (http://www.badd-cao.net:2345/search, pubchem ID: 5281767) (18) and Swiss Target Prediction database (STD, http://www.swisstargetprediction.ch/) (19) were used to capture targets of curcumin. All targets of curcumin from HIT 2.0 and STD databases were regarded as potential drug targets.

### Establishment of potential NSCLC-associated targets

Conceivable NSCLC-related targets were obtained from Comparative Toxicogenomics Database (CTD, http://ctdbase.org/) and the DisGeNET Database (https://www.disgenet.org/home/) by using the search term ‘non-small cell lung carcinoma'. According to experience, the criteria for selecting potential targets are inference score ≥40 in the CTD database and score_gda ≥0.3 in the DisGeNET database (20,21).

### Construction of NSCLC-related targets of curcumin

We screened the curcumin targets and NSCLC-associated proteins using the Venn diagram web tool (https://hiplot.com.cn/cloud-tool/drawing-tool/list), and common targets were identified as NSCLC-associated curcumin targets for the bioinformatics analysis.

### PPI enrichment and cluster analyses of common targets

To investigate the interactions between curcumin targets and NSCLC targets, we input the intersection target genes into the interaction database platform STRINGv.11.5 (https://string-db.org/), and a protein-protein interaction (PPI) network was acquired, with the settings of Organism for “*Homo sapiens*” and a confidence score ≥0.4. We also investigated potential clusters in 67 hub genes between curcumin and NSCLC groups utilizing MCODE in Cytoscape. MCODE is the Cytoscape plugin for clustering a given network according to its topology to determine densely related regions, which can be utilized to identify hub genes. In our study, the degree cutoff and k-core threshold were set to 2 in MCODE parameters.

### Gene Ontology functional analysis

The DAVID Knowledgebase (https://david.ncifcrf.gov/home.jsp) has a comprehensive annotation function, and the common genes between curcumin and NSCLC groups were imported into the database for bioinformatics analysis, including Gene Ontology (GO) analyses and Kyoto Encyclopedia of Genes and Genomes (KEGG) pathway annotations. We performed an enrichment analysis and explored the main biological processes (BPs), cellular components (CCs), and molecular functions (MFs). The top 10 hub genes in each category were selected for further analysis. For inclusion, we set a P-value <0.05.

### Molecular docking

Based on the enrichment analysis of potential clusters, the central NSCLC-associated genes targeted by curcumin were retrieved. These targets were affirmed via molecular docking (Discovery Studio 2019 version). We downloaded the 3D structure of the selected hub protein (STAT3, PDB ID: 6NUQ; AKT1, PDB ID: 7NH5; MAPK3, PDB ID: 4QTB; HIF1A, PDB ID: 3KCY; JUN, PDB ID: 2P33; and EGFR, PDB ID: 5XDK) from RSCB PDB database (https://www.rcsb.org/) in pdb format. The proteins were prepared for docking by removing water and hetero-atoms from PDB file of proteins in the Discovery Studio software, while polar hydrogen was added to proteins and ligands. The potentially active pocket is then constructed, namely ligand-binding sites, by clicking “Define and Edit Binding Site” module in the protein, and the original ligand is deleted.

Discovery Studio was used to reveal cross-talk between top-ranked curcumin and residues of the selected hub protein to elucidate 2D and 3D views of their docking positions.

### Cell cultures and administration

Human lung cancer lines A549 and H460 were purchased from American Type Culture Collection (ATCC) and cultured in DMEM medium, which contained 10% fetal bovine serum and 100 U/mL penicillin/streptomycin under a humidified atmosphere at 37°C with 5% CO_2_. Then, the cells were treated with 10 μM curcumin for 24 and 48 h.

### Real-time PCR

mRNA was extracted from each sample and reverse-transcribed into cDNA, according to PrimeScript RT-PCR-Kit (Takara Bio, Japan). The total volume of the reaction system was 20 μL. The reaction conditions were pre-incubation at 95°C for 30 s and one cycle, followed by denaturation at 95°C for 10 s, and elongation at 60°C for 30 s, repeated for 45 cycles. Each sample was run in triplicate and averaged. The relative gene expression was calculated by the 2^-△△^Ct method. All the primers used are displayed in Table 1.

**Table 1:** Primers used for real-time PCR.

Species/Gene	Accession numbers	Forward primer and reverse primer (5′-3′)
*Hs HIF1A*	NM_001243084.2	F: GAACGTCGAAAAGAAAAGTCTCG
		R: CCTTATCAAGATGCGAACTCACA
*Hs CCND1*	NM_053056.3	F: GCTGCGAAGTGGAAACCATC
		R: CCTCCTTCTGCACACATTTGAA
*Hs AKT1*	NM_001382431.1	F: AGCGACGTGGCTATTGTGAAG
		R: GCCATCATTCTTGAGGAGGAAGT
*Hs STAT3*	NM_001384993.1	F: CAGCAGCTTGACACACGGTA
		R: AAACACCAAAGTGGCATGTGA
*Hs IL-6*	NM_001371096.1	F: ACTCACCTCTTCAGAACGAATTG
		R: CCATCTTTGGAAGGTTCAGGTTG
*Hs Caspase-3*	NM_032991.3	F: CATGGAAGCGAATCAATGGACT
		R: CTGTACCAGACCGAGATGTCA
*Hs rps16*	NM_001020	F: TGGTCTCATCAAGGTGAACG
		R: AAGTGAGTTTTGAGTCACGA

### Statistical analysis

The data are reported as means±SD and were analyzed with Graphpad Prism 9.0 (USA). Statistical comparisons between the lung cancer group and the curcumin-treated group were done with one-way ANOVA with Dunnett's multiple comparison test. P<0.05 was considered statistically significant.

## Results

### Establishment of common targets for curcumin and NSCLC

**Figure 2:**
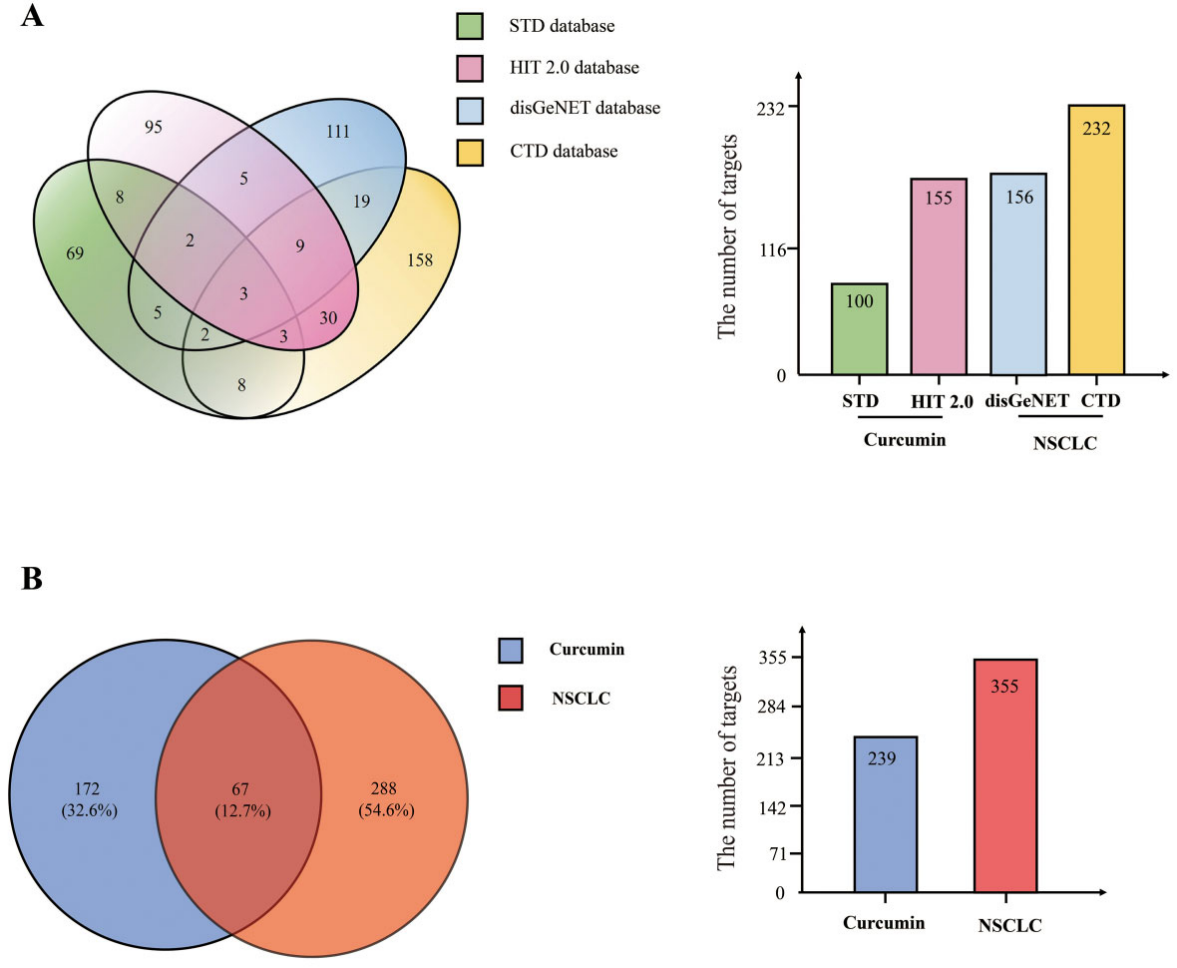
**A**, Targets of curcumin for treatment of non-small cell lung cancer (NSCLC). **B**, Targets of curcumin and NSCLC. A total of 67 genes (12.7%) are common to curcumin and NSCLC lists.

A total of 155 curcumin targets were identified based on HIT 2.0 (Supplementary Table S1), and 100 curcumin-related targets were retrieved from the STD database (Supplementary Table S2). In addition, we captured 156 and 232 NSCLC-related targets from the DisGeNET (Supplementary Table S4) and CTD databases (Supplementary Table S3), respectively (Figure 2A). After removing the duplicate targets, we obtained 239 targets of cucumin and 355 targets of NSCLC (Figure 2B). Subsequently, 67 common targets between curcumin and NSCLC were obtained, as shown in Figure 2.

### GO and KEGG analyses of disease targets

**Figure 3:**
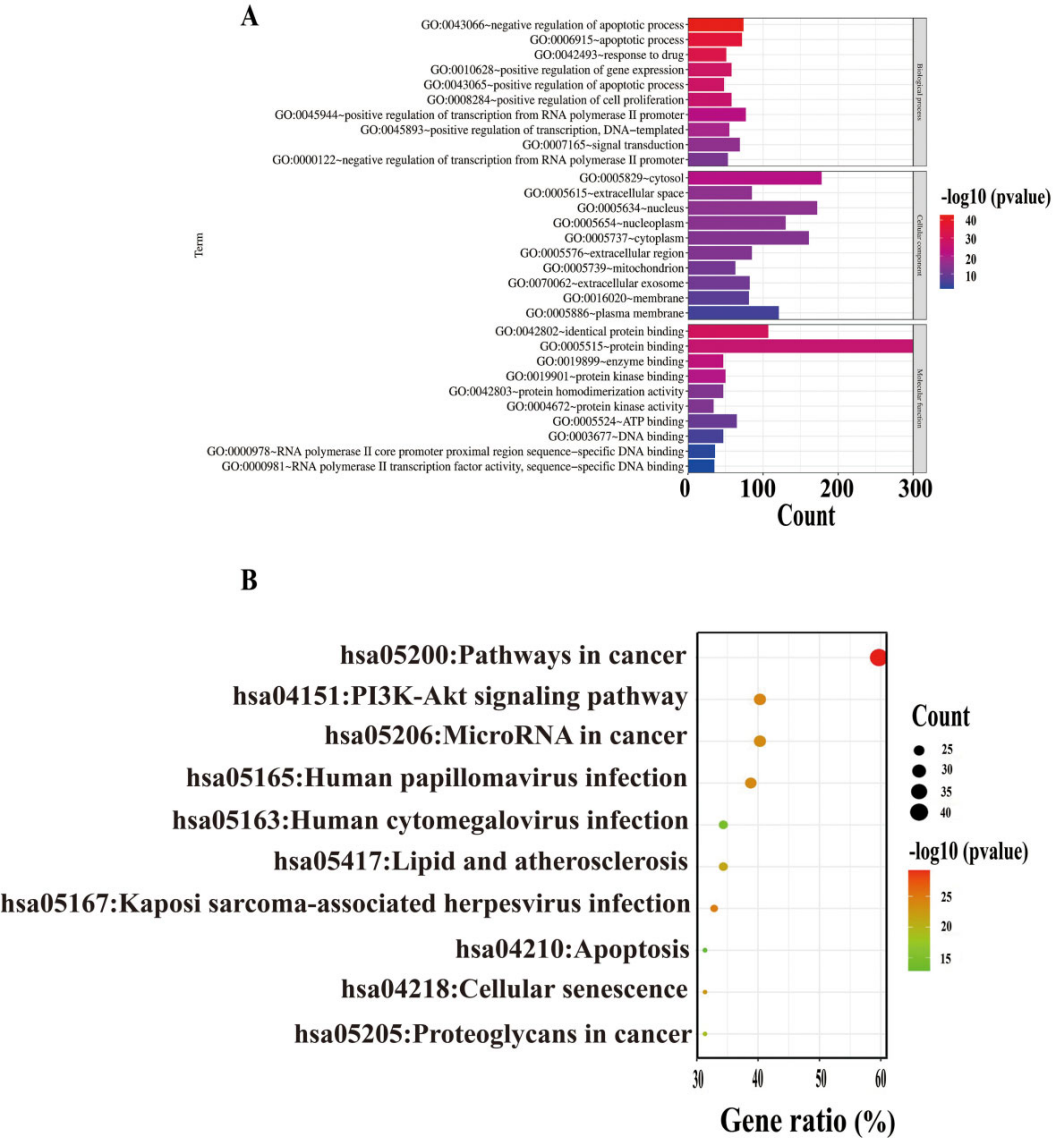
**A**, Gene Ontology (GO) and Kyoto Encyclopedia of Genes and Genomes (KEGG) analysis of non-small cell lung cancer (NSCLC)-related genes. **B**, The top 10 significantly enriched terms in biological processes, cellular components, and molecular functions of the KEGG database.

We completed the KEGG and GO enrichment analyses of the NSCLC 355 genes mentioned above. The GO enrichment analysis revealed that the BPs of the NSCLC targets were mainly associated with the apoptotic process, response to a drug, gene expression, and cell proliferation. The MFs of the NSCLC targets were related to protein binding, enzyme binding, and protein kinase binding and activity. The CCs relevant to NSCLC were activated in the cytosol, extracellular space, nucleus, nucleoplasm, or cytoplasm (Figure 3A). The KEGG pathway analysis showed that the main pathways related to NSCLC were cancer pathways, the PI3K-AKT signaling pathway, microRNAs related to cancer, apoptosis, cellular senescence, and proteoglycans in cancer (Figure 3B).

### PPI network analysis of common targets

**Figure 4:**
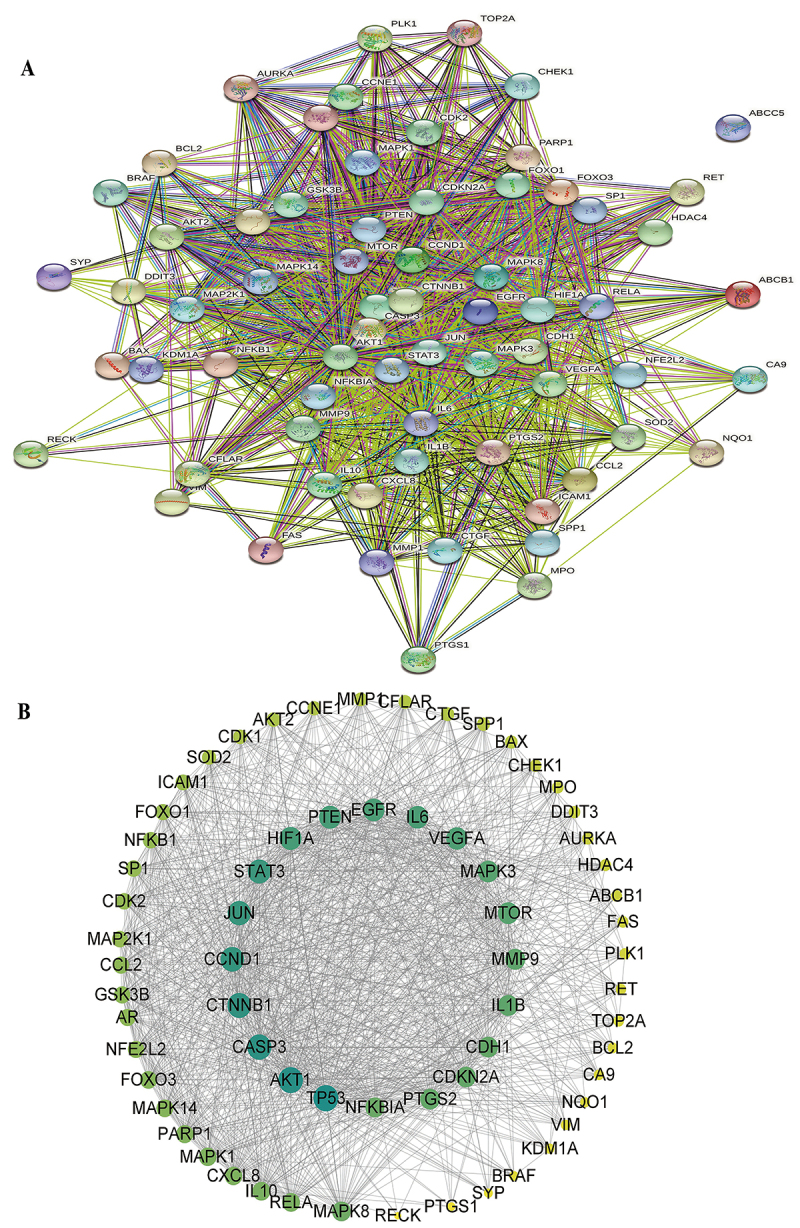
**A**, Protein-protein interaction (PPI) network of targets for treating non-small cell lung cancer (NSCLC). **B**, Putative targets are arranged clockwise on the basis of the degree value from large to small.

A PPI network analysis of the 67 common targets was performed using the STRING database online service platform, as shown in Figure 4A. The network included 67 nodes and 1111 edges. We imported the results of the STRING analysis into Cytoscape according to the degree of interaction, and this contributed to a total of 66 nodes and 1111 edges, with an average number of 33,667 neighbors, a diameter of 3, and a clustering coefficient of 0.793 (Figure 4B)

### GO and KEGG analyses of common targets

**Figure 5:**
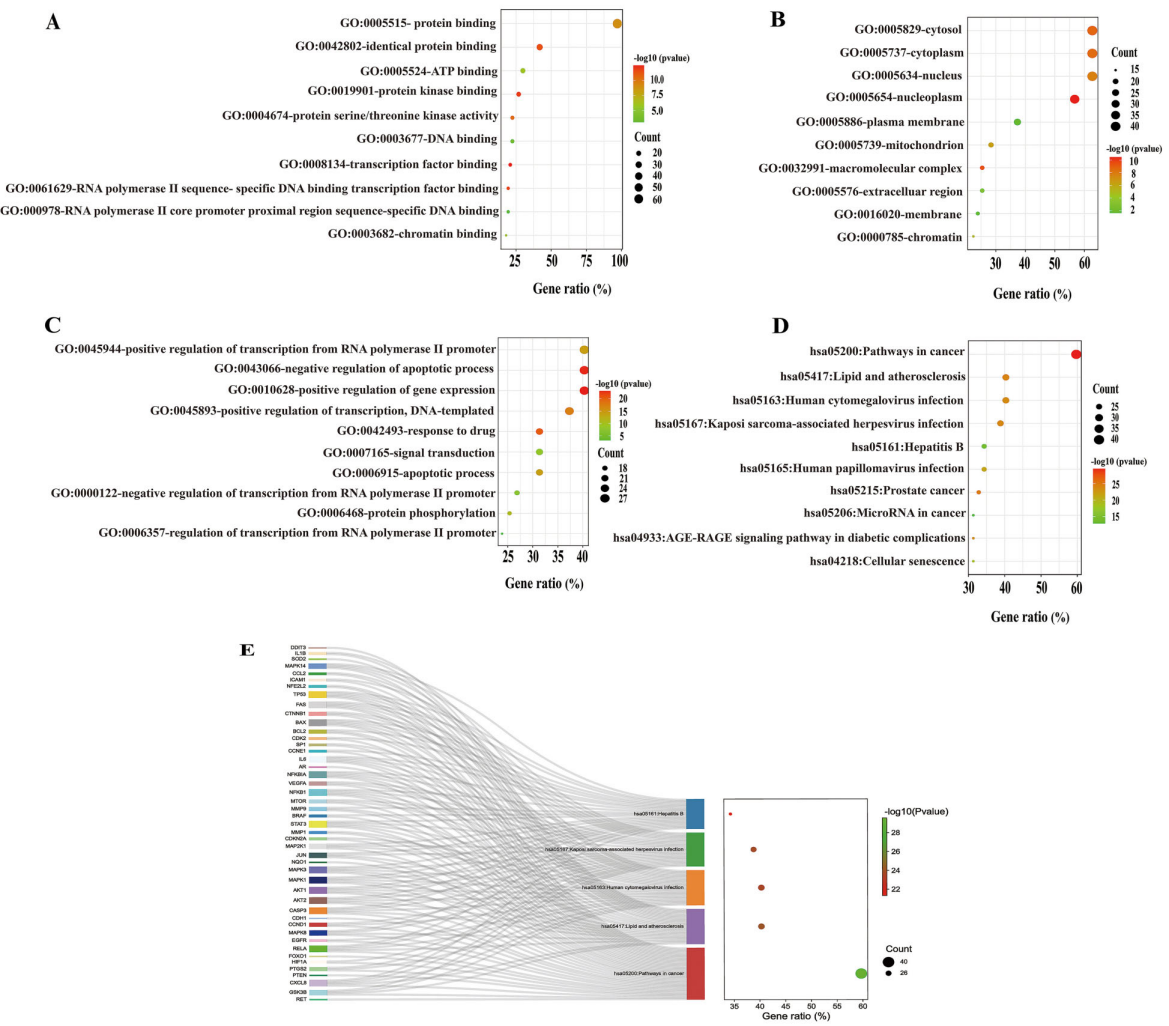
Gene Ontology (GO) and Kyoto Encyclopedia of Genes and Genomes (KEGG) analysis of hub targets. **A**, Top 10 significantly enriched terms in molecular functions (MFs). **B**, Top 10 significantly enriched terms in cellular components (CCs). **C**, Top 10 significantly enriched terms in biological processes (BPs). **D**, The top 10 pathways of co-targets based on KEGG enrichment analysis. **E**, Hub genes analysis of top 5 significantly signaling pathways.

The DAVID platform was used for the GO function enrichment and KEGG analysis of the 66 pivotal targets. A total of 491 GO terms involving 376 BPs, 48 CCs, 67 MFs, and 150 KEGG pathways were enriched. The 10 most significantly enriched terms for the MFs (Figure 5A), CCs (Figure 5B), BPs (Figure 5C), and KEGG pathways (Figure 5D) were selected for a visual analysis according to the gene counts and P-value (P<0.05). The BPs mainly included regulation of gene expression, positive regulation of transcription, regulation of transcription from the RNA polymerase II promoter, response to a drug, apoptotic process, signal transduction, and protein phosphorylation. The CCs were the cytosol, cytoplasm, nucleus, nucleoplasm, plasma membrane, mitochondrion, macromolecular complex, extracellular region, membrane, and chromatin. The MFs mainly involved protein binding, identical protein binding, ATP binding, protein kinase binding, protein serine/threonine kinase activity, DNA binding, transcription factor binding, RNA polymerase II sequence-specific DNA binding transcription factor binding, RNA polymerase II core promoter proximal region sequence-specific DNA binding, and chromatin binding. In addition, NSCLC treatment by curcumin may be mainly related to pathways in cancer, prostate cancer, microRNAs in cancer, cellular senescence, and other signaling pathways. Furthermore, we obtained the core genes from the top 5 significant signaling pathways (Figure 5E).

### Cluster analysis of common targets

**Figure 6:**
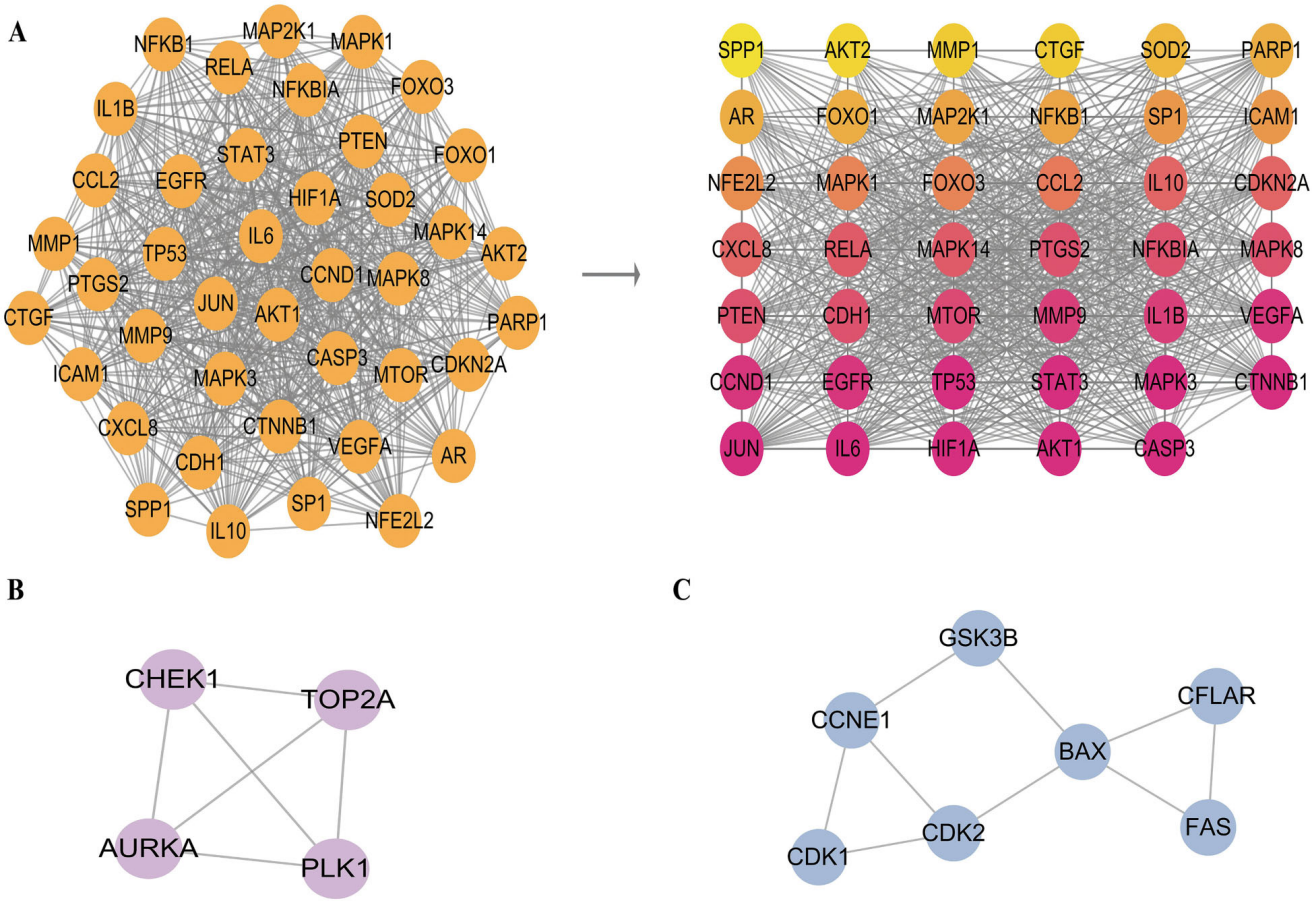
The three significant clusters within protein-protein interaction (PPI) network construction. **A**, Cluster 1 with 41 nodes. **B**, Cluster 2 with 4 nodes. **C**, Cluster 3 with 7 nodes.

To classify and verify the potential clusters of the curcumin targets for treating NSCLC within this network, the MCODE plugin was used to assess 3 clusters (shown in Table 2) depending on the significance and degree of interaction, and these were sorted via scores. Cluster 1 had 41 nodes and 690 edges, and the score was 34.5, of which the seed was nuclear factor erythroid 2-related factor 2 (NFE2L2, NRF2) (Figure 6A). Cluster 2 involved 4 nodes with 6 edges, with a score of 4 (Figure 6B). The seed for cluster 2 was the serine/threonine-protein kinase Chk1 (CHEK1), which is required for checkpoint-mediated cell cycle arrest and activation of DNA repair in response to the presence of DNA damage or unreplicated DNA. Cluster 3 included 7 nodes and 9 edges, with a score of 3 (Figure 6C), and the seed was CASP8 and FADD-like apoptosis regulator (CFLAR), which is a crucial link between cell survival and cell death pathways in mammalian cells.

**Table 2:** The 52 genes with degree ≥2 were divided into 3 cluster subnetworks in 66 target genes.

Gene ID	Full name	Degree	MCODE score
**Cluster 1**			
*CASP3*	Caspase-3	40	23.22617354
*CTNNB1*	Catenin beta-1	40	23.22617354
*JUN*	Transcription factor Jun	40	23.22617354
*IL6*	Interleukin-6	40	23.22617354
*MAPK3*	Mitogen-activated protein kinase 3	40	23.42342342
*HIF1A*	Hypoxia-inducible factor 1-alpha	40	23.22617354
*STAT3*	Signal transducer and activator of transcription 3	40	23.22617354
*AKT1*	Non-specific serine/threonine protein kinase	40	23.22617354
*TP53*	Tumor protein p53	40	23.22617354
*CCND1*	G1/S-specific cyclin-D1	39	23.81269841
*VEGFA*	Vascular endothelial growth factor A	39	23.98991597
*EGFR*	Epidermal growth factor receptor	39	24.84677419
*MMP9*	Matrix metalloproteinase-9	38	24.3315508
*IL1B*	Interleukin-1 beta	38	23.42342342
*MTOR*	Serine/threonine-protein kinase mTOR	37	23.81269841
*NFKBIA*	NF-kappa-B inhibitor alpha	36	23.42342342
*PTGS2*	Prostaglandin G/H synthase 2	36	23.26203209
*PTEN*	Phosphatidylinositol 3,4,5-trisphosphate 3-phosphatase and dual-specificity protein phosphatase	36	23.98991597
*MAPK8*	Mitogen-activated protein kinase 8	36	24.84677419
*CDH1*	Cadherin-1	36	23.57954545
*MAPK14*	Mitogen-activated protein kinase 14	35	23.21746881
*RELA*	Transcription factor RelA	35	23.42342342
*CXCL8*	Interleukin-8	34	24.92877493
*IL10*	Interleukin-10	34	24.92877493
*CDKN2A*	Cyclin-dependent kinase inhibitor 2A	34	23.26203209
*CCL2*	C-C motif chemokine 2	32	24.92877493
*FOXO3*	Forkhead box protein O3	31	22.60645161
*MAPK1*	Mitogen-activated protein kinase 1	31	23.58870968
*NFE2L2*	Nuclear factor erythroid 2-related factor 2	30	25.0
*SP1*	Transcription factor Sp1	29	24.0
*ICAM1*	Intercellular adhesion molecule 1	29	23.0
*NFKB1*	Nuclear factor NF-kappa-B p105 subunit	28	20.28045977
*MAP2K1*	Dual specificity mitogen-activated protein kinase kinase 1	28	23.65811966
*PARP1*	Poly [ADP-ribose] polymerase 1	27	21.01058201
*AR*	Androgen receptor	27	22.14814815
*FOXO1*	Forkhead box protein O1	27	21.59384615
*SOD2*	Superoxide dismutase [Mn], mitochondrial	26	20.91699605
*CTGF*	Cellular communication network factor 2	24	22.0
*MMP1*	Matrix metalloproteinase-1	24	21.70666667
*AKT2*	RAC-beta serine/threonine-protein kinase	23	22.0
*SPP1*	Secreted phosphoprotein 1	22	22.0
**Cluster 2**			
*TOP2A*	DNA topoisomerase 2-alpha	3	11.11764706
*AURKA*	Aurora kinase A	3	12.23529412
*PLK1*	Serine/threonine-protein kinase Plk1	3	12.33088235
*CHEK1*	Serine/threonine-protein kinase Chk1	3	13.37662338
**Cluster 3**			
*BAX*	Apoptosis regulator Bax	4	15.89542484
*CDK2*	Cyclin-dependent kinase 2	3	19.33333333
*CCNE1*	G1/S-specific cyclin-E1	3	15.96969697
*GSK3B*	Glycogen synthase kinase-3 beta	2	19.39655172
*FAS*	Fatty acid synthase	2	16.0
*CDK1*	Cyclin-dependent kinase 1	2	17.57142857
*CFLAR*	CASP8 and FADD-like apoptosis regulator	2	19.68379447

### Molecular docking results and analysis

Based on Table 2, the top 12 targets were CASP3, CTNNB1, JUN, IL6, MAPK3, HIF1A, STAT3, AKT1, TP53, CCND1, VEGFA, and EGFR. We selected six potential targets (STAT3, AKT1, MAPK3, HIF1A, JUN, and EGFR) for molecular docking with curcumin (shown in Supplementary Figure S1). After the simulation analysis, the parameters including the absolute energy, the amount of docking poses (Conf Number) and the relative energy, and the overall score of Libdock (LibDock score) were acquired. Lower absolute and relative energy between the inhibitor and the docking site suggested a stronger repression of the target protein by the inhibitor, and a higher Conf number showed a higher likelihood of interaction between the inhibitor and the site. Finally, a higher LibDock score meant a stronger suppression of the inhibitor. The binding data of curcumin with the hub targets are shown in Table 3. In addition, according to the LibDock score, the targets ranking from high to low were HIF1A, MAPK3, AKT1, JUN, EGFR, and STAT3. Therefore, HIF1A, MAPK3, and AKT1 may be the vital binding ligands of targets relevant to the therapeutic effect of curcumin for NSCLC.

**Table 3:** Docking parameters of target proteins and curcumin.

Core target	PDB ID	Absolute energy	Conf number	Relative energy	LibDock score
HIF1A	3KCY	82.2212	153	9.31473	101.966
MAPK3	4QTB	81.5794	135	8.67292	101.291
AKT1	7NH5	83.8221	172	10.9156	97.8572
JUN	2P33	78.9721	82	6.0656	81.1181
EGFR	5XDK	75.3712	18	2.46476	65.5782
STAT3	6NUQ	85.9268	198	13.0203	37.9724

### Curcumin inhibited levels of hub genes in A549 cells

**Figure 7:**
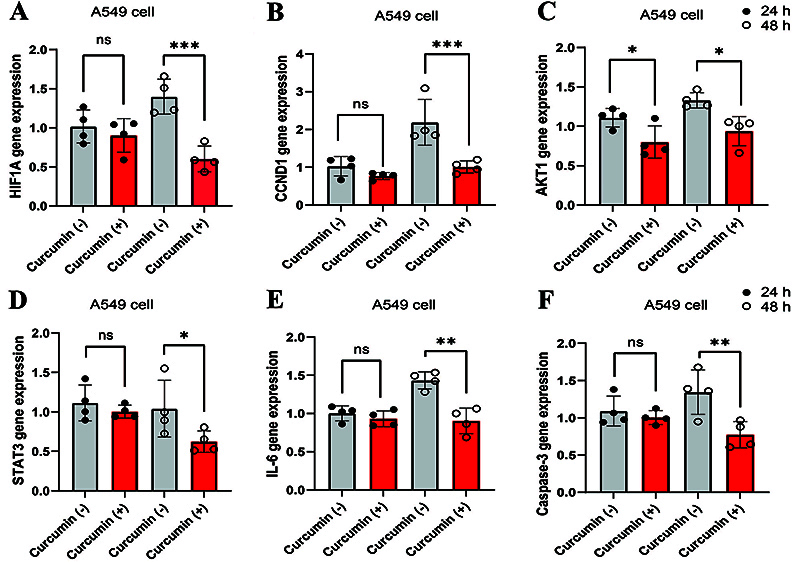
**A-F**, Curcumin reduced the gene expressions of *HIF1A*, *CCND1*, *AKT1*, *STAT3*, *IL-6*, and *caspase-3* in A549 cells. Data are reported as means±SD (n=4). *P<0.05, **P<0.01, and ***P<0.001 (ANOVA); ns: not significant.

Compared with A549 cells, the levels of *HIF1A*, *CCND1*, *AKT1*, *STAT3*, *IL-*6, and *caspase-3* genes were significantly reduced in the 48-h curcumin treatment group (Figure 7A-F). However, in the 24-h curcumin treatment group, only *AKT1* gene expression was statistically significant.

### Curcumin inhibited levels of hub genes in NIC-H460 cells

**Figure 8:**
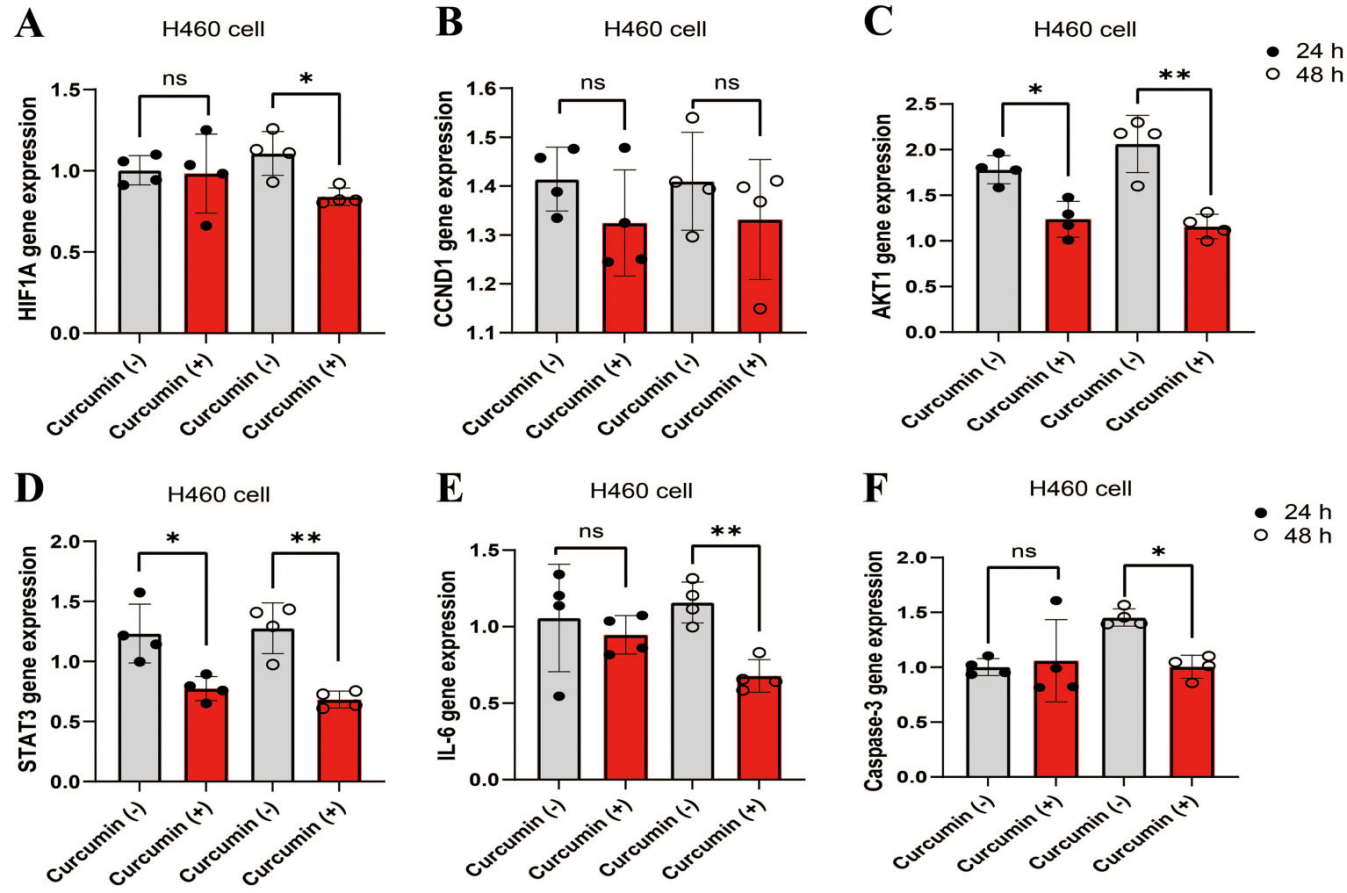
**A-F**, Curcumin suppressed the gene expressions of *HIF1A*, *AKT1*, *STAT3*, *IL-6*, and *caspase-3* in H460 cells. Data are reported as means±SD (n=4). *P<0.05 and **P<0.01 (ANOVA); ns: not significant.

Compared with the H460 group, gene expression was significantly down-regulated in the 48-h curcumin-treated group, except for the *CCND1* gene (Figure 8A-F). Interestingly, in the 24-h curcumin treatment group, *AKT1* and *STAT3* gene expressions were statistically significant, but other gene levels were not.

## Discussion

Over the years, network pharmacology has been considered an essential tool for identifying potential targets for drug therapies, including the unexpected COVID-19 events (22). In this study, a strategy, based on network pharmacology combined with molecular docking, was performed to reveal the molecular mechanism of curcumin for the treatment of NSCLC. We obtained 67 common targets between curcumin and NSCLC by screening public databases. The GO enrichment analysis, including BPs, CCs, and MFs, explained the function of the protein targets, and the KEGG pathway enrichment analysis suggested that 67 target proteins significantly enriched 150 signal pathways, including cancer-related pathways, such as the PI3K-AKT signaling pathway, Foxo signaling pathway, microRNAs in cancer, MAPK signaling pathway, HIF-1 signaling pathway, etc. In addition, the PPI network and cluster analysis indicated that CASP3, CTNNB1, JUN, IL6, MAPK3, HIF1A, STAT3, AKT1, TP53, CCND1, VEGFA, and EGFR may be vital to the effect of curcumin against NSCLC.

Of course, network pharmacology screening also has limitations, such as screening criteria, scoring of signaling pathways, and database integration, etc. In this manuscript, we integrated multiple databases on drug targets and NSCLC targets, and obtained the final pivotal targets through the screening standards and methods, as well as molecular docking, provided by multiple literature. In addition, the effectiveness of the targets was also verified through *in vitro* experiments. These data reliably support that curcumin passes through these key targets and thus treats NSCLC.

### Curcumin inhibited inflammation

The functional inactivation or mutation of *TP53* (tumor suppressor gene) can contribute to impairing epithelial function and promoting the generation of lung cancer, including NSCLC and SCLC (23,24). These alterations not only trigger immune escape (25), but also elevate the release of inflammatory cytokines (IL-6, IL-10, and TNF-α) (26). In return, the inflammatory response increases the production of cancer (27). Although the mechanism of inflammation-induced cancer is not fully elucidated, two related hypotheses have emerged, including an intrinsic pathway (activating oncogenes or inactivating tumor suppressor genes) and an extrinsic pathway (chronic inflammatory diseases) (28). Research shows that the dysregulation of the IL-6/STAT3 signaling pathway mediates lung cancer cell proliferation and invasion, and the suppression of the IL-6/STAT3 pathway prevents the progression of lung cancer (29). Furthermore, blocking the IL-6/STAT3 pathway improves cachexia (30). In addition to the IL-6/STAT3 pathway, the MAPK/ERK and PI3K/AKT pathways are the classical carcinogenic approaches involved in controlling tumorigenesis and development (31). They not only regulate the self-renewal activity and inflammation of cancer stem cells (32), but also result in drug resistance in lung cancer. It is worth noting that although endogenous and exogenous pathways are mediated by distinct molecular mechanisms, these two pathways collectively contribute to increased cell proliferation and inhibition of apoptosis, angiogenesis, and apoptosis, and extracellular matrix remodeling, migration, and invasion by activating key transcription factors, such as NF-κB, STAT3, and HIF1A in tumor cells (33). In brief, preclinical evidence reveals that targeting IL-6, STAT3, MAPK3, or AKT1 provides an effective way to suppress lung cancer. The bioinformatics analysis revealed that curcumin inhibited the expression of genes related to these factors and disturbed mutations in TP53, VEGFA, and EGFR to attenuate NSCLC.

### Curcumin regulated cell apoptosis in NSCLC

Caspase-3, known as an executioner caspase, exhibits a dominant role in apoptosis and becomes a critical target for natural or synthetic compounds in cancer therapy (34). This enzyme is an inactive zymogen in cells and does not execute apoptosis until it is cleaved by initiator caspases (35). The process of cell apoptosis can be roughly divided into the following stages: receiving apoptotic signals → the interaction between apoptotic regulatory molecules → the activation of proteolytic enzymes (caspase) → entering a continuous reaction process. Diverse external factors trigger apoptosis in different ways and cause various signal transductions. Therefore, regulating the expression of caspase-3 or activated caspase-3 affects lung cancer apoptosis (36).

### Curcumin regulated cell cycle in NSCLC

Cyclin Ds, including D1, D2, and D3, control cell cycle progression by mediating cell proliferation and extracellular stimulation, of which cyclin D1 (CCND1) is more attractive because of its widespread dysregulation in human cancer (37). It is not a secret that a cyclin D imbalance directly results in neoplastic growth. Numerous studies illustrate that elevating CCND1 expression promotes the proliferation progress of NSCLC (38), and inhibiting CCND1 suppresses cell proliferation, migration, and invasion and induces apoptosis in NSCLC (39). In addition, downregulating CCND1 expression is beneficial to enhance the toxicity and sensitivity of some chemotherapeutic drugs (5-FU, gefitinib, cisplatin) to drug-resistant NSCLC (40). Therefore, targeting CCND1 provides an effective approach to preventing NSCLC.

One limitation of this study was that animal experiments were not completed. The exact target of curcumin for NSCLC could be further refined and elucidated under *in vivo* conditions. In addition, the conditions of *in vitro* experiments are variable and can bias the results. Subsequently, we will carry out animal experiments to verify and improve the results. However, it must be acknowledged that data from *in vitro* experiments are a powerful guarantee for the success of animal experiments.

### Conclusion

In this study, curcumin was identified as a potential effective active agent against NSCLC by controlling multiple pivotal targets. The pharmacological effect of curcumin against NSCLC may be linked with the regulation of CCND1, CASP3, HIF1A, IL-6, MAPK3, STAT3, AKT1, and TP53 targets. The specific pathways were the cancer pathway, MAPK signaling pathway, Foxo signaling pathway, microRNAs in cancers, and HIF1A signaling pathway. Our data provided a comprehensive perspective for accelerating clinical or preclinical studies for the combined use of curcumin and its mechanism in treating NSCLC.

## Supplementary Material

Click to view [zip].
