# Supplementary material for: Decoding potential targets and pharmacologic mechanisms of curcumin in treating non-small cell lung carcinoma via bioinformatics and molecular docking
Source: Braz J Med Biol Res. 2024 Sep 6;57:e13550. doi: 10.1590/1414-431X2024e13550 (PMC11379430; doi:10.1590/1414-431X2024e13550)
Supplement: Supplementary file 1 [file 1414-431X-bjmbr-57-e13550-suppl.zip › 13550_Figure_S1.pdf]

Figure S1. Molecular docking (3D and 2D) of curcumin with key targets. **A**, HIF1A, hydrogen bonds: TYR145 and GLN 239. **B**, MAPK3, hydrogen bonds: ALA52, LYS71, THR85, and ASP184. **C**, AKT1, hydrogen bonds: LYS179, THR211, and GLY294. **D**, JUN, hydrogen bonds: LYS93 and ASN152. **E**, EGFR, hydrogen bonds: MET793, PHE795, CYS797, and TYR801. **F**, STAT3, hydrogen bonds: ASP661.

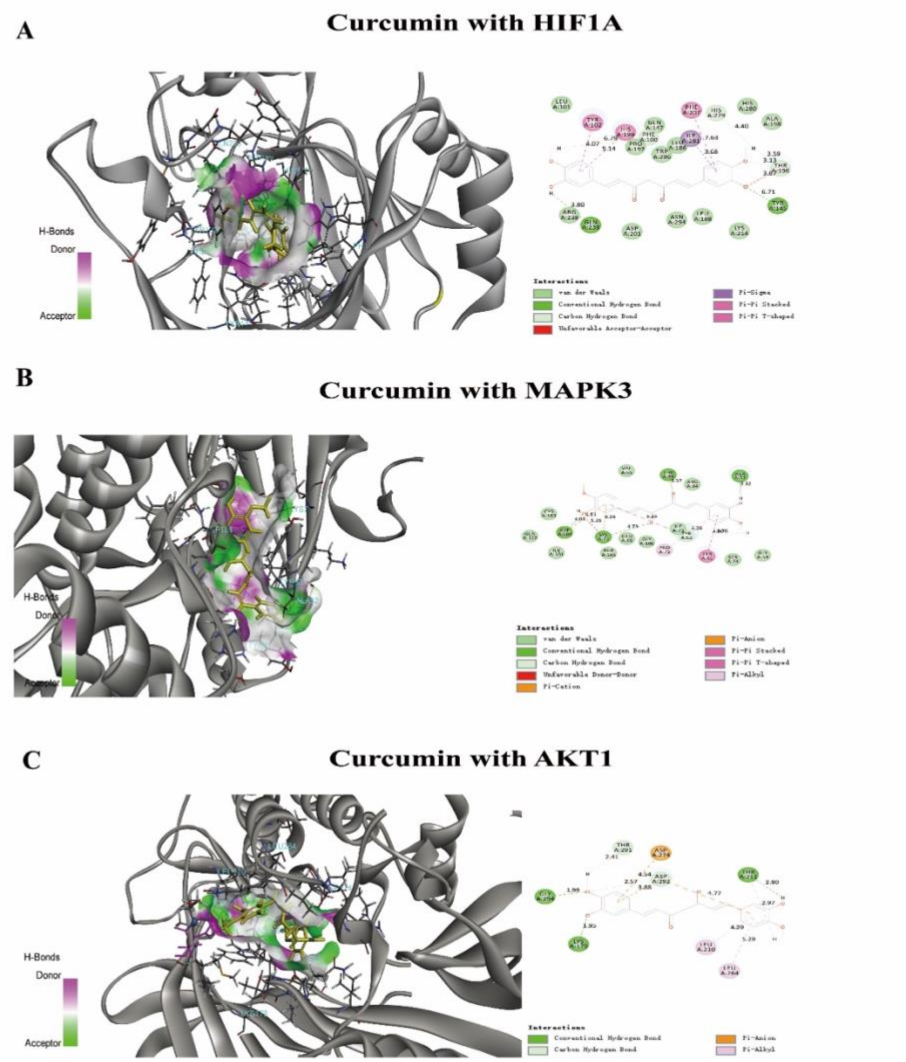

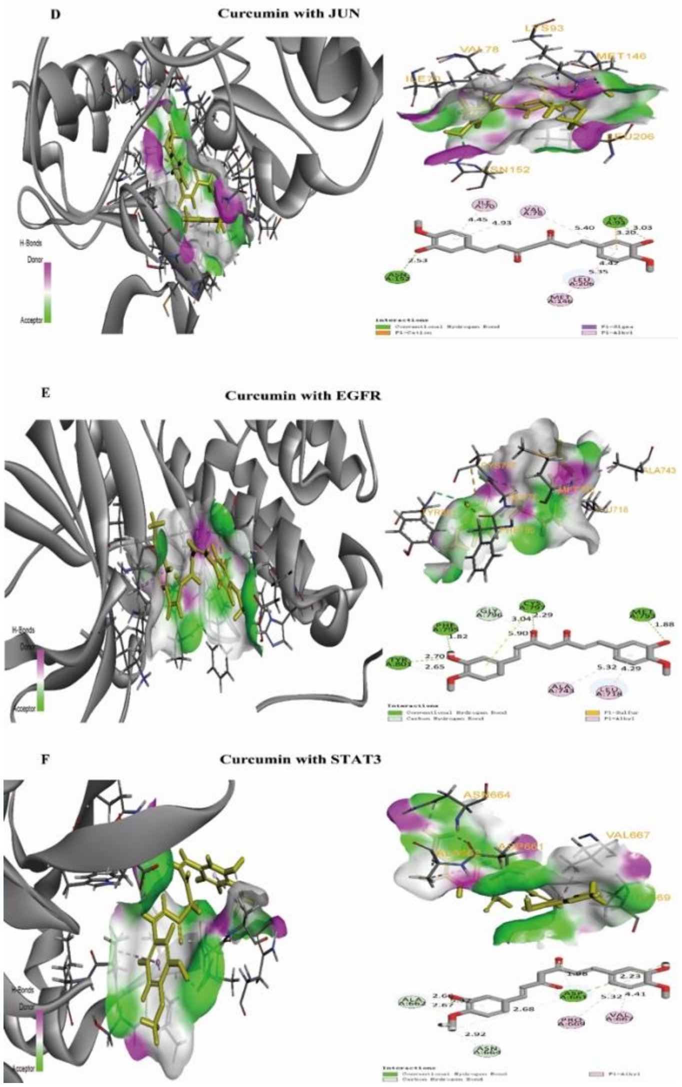
D
Curcumin with JUN
VAL78
MET146
H-Bonds
Donor
Acceptor
E
Curcumin with EGFR
ALA743
H-Bonds
Acceptor
F
Curcumin with STAT3
VAL667
Acceptor
